# Assessment of the effect of left atrial cryoablation enhanced by ganglionated plexi ablation in the treatment of atrial fibrillation in patients undergoing open heart surgery

**DOI:** 10.1186/s13019-017-0625-1

**Published:** 2017-08-17

**Authors:** Jiří Bárta, Radim Brát

**Affiliations:** Cardiac Surgery, University Hospital Ostrava, 17.listopadu 1790, 708 52 Ostrava-Poruba, Czech Republic

**Keywords:** Atrial fibrillation, Left atrial cryoablation, Ganglionated plexi, Open-heart surgery

## Abstract

**Background:**

The aim of our study was to investigate, whether enhancement of left atrial cryoablation by ablation of the autonomic nervous system of left atrium leads to influencing the outcomes of surgical treatment of atrial fibrillation in patients with structural heart disease undergoing open-heart surgery.

**Methods:**

The observed patient file consisted of 100 patients, who have undergone a combined open-heart surgery at our department between July 2012 and December 2014. The patients were indicated for the surgical procedure due to structural heart disease, and suffered from paroxysmal, persistent, or long-standing persistent atrial fibrillation. In all cases, left atrial cryoablation was performed in the extent of isolation of pulmonary veins, box lesion, connecting lesion with mitral annulus, amputation of the left atrial appendage and connecting lesion of the appendage base with left pulmonary veins. Furthermore, 35 of the patients underwent mapping and radiofrequency ablation of ganglionated plexi, together with discision and ablation of the ligament of Marshall (Group GP). A control group was consisted of 65 patients without ganglionated plexi intervention (Group LA). The main primary outcome was establishment and duration of sinus rhythm in the course of one-year follow-up.

**Results:**

Evaluation of the number of patients with a normal sinus rhythm in per cent has shown comparable values in both groups (Group GP - 93.75%, Group LA – 86.67%, *p* = 0.485); comparable results were also observed in patients with normal sinus rhythm without anti-arrhythmic treatment in the 12th month (Group GP – 50%, Group LA – 47%, *p* = 0.306). We have not observed any relation between the recurrence of atrial fibrillation and the presence of a mitral valve surgery, or between the presence of a mitral and tricuspid valves surgery and between the left atrial diameter > 50 mm.

**Conclusions:**

Enhancement of left atrial cryoablation by gangionated plexi ablation did not influence the outcomes of surgical ablation due to atrial fibrillation in our population in the course of 12-month follow-up.

**Trial registration:**

The study was approved retrospectively by the Ethics Committee of the University Hospital Ostrava (reference number 867/2016).

## Background

The growing incidence of atrial fibrillation (AF) in the population [1] and its clinical [1] and economical [2] importance supports the progressive growth of research of pathophysiology of atrial fibrillation and factors influencing the initiation and maintenance of this most frequently observed heart arrhythmia in the last decades, which is reflected also in the frequency of updates of Guidelines for treatment of AF issued by various cardiology and heart surgery expert societies.

One of the main factors playing an important role in the initiation and duration of atrial fibrillation is the Autonomic Nervous System (ANS) [3–5]. ANS may be divided into two main components – extrinsic and intrinsic [6]. Extrinsic ANS has a sympathetic and parasympathetic branch. Sympathetic fibres originate from the ganglia along cervical and thoracic spine, and end at the heart surface. Parasympathetic fibres originate in nucleus ambigus in medulla oblongata, and continue as a part of nervus vagus; most of them terminate in the adipose tissue (fat pad) between the superior vena cava and the aorta [7]. Intrinsic ANS is formed with a number of heart ganglia, containing approximately 200–1000 neurons, and synapses of sympathetic and parasympathetic fibres [8, 9]. Most of the ganglia are organized in ganglionated plexi (GP), embedded in epicardial fat pads on the surface of atria and ventricles. They participate upon the interconnection and modulation of interactions between extrinsic and intrinsic ANS [10], and, apart from others, influence the function of the sinoatrial and atrioventricular node [11, 12].

Already in 1978, Coumel et al. [13] in their work pointed out the fact that under certain conditions, the activity of intrinsic ANS may cause paroxysm of atrial arrhythmia. Other works looking into with a more detailed study of intrinsic ANS show the possibility of evocation of rapid trigger activity with stimulation of intrinsic ANS, with electric impulses [14], as well as administration of autonomous neurotransmitters [15–17]. This trigger activity is most frequently observed in myocardial sleeves of the pulmonary veins [18]. The mechanism behind the initiation of trigger activity is a vegetative influencing of the intracellular level of calcium ions, and the length of duration of the action potential by causing early after depolarisations [19]. Apart from this, further changes of autonomous regulation (autonomous remodelling) may, in interaction with other AF mechanisms (with structural remodelling) lead to a formation of a substrate, which is so important for maintenance of AF [20]. Clinical trials dealing with catheterization therapy of AF present a significant improvement of the outcomes in cases of enhancement of the catheterization ablation by GP ablation [21–23]. In accordance with the outcomes of these studies, also other outcomes of surgical treatment of lone AF via minimally invasive surgical approach are presented [24–26]. The aim of our study was to investigate, whether enhancement of left atrial cryoablation by ablation of the autonomic nervous system of left atrium leads to influencing the outcomes of surgical treatment of atrial fibrillation in patients with structural heart disease undergoing open-heart surgery.

## Methods

### Patient selection

The our patient population consisted of 1 hundred subjects, who underwent an open-heart surgery between July 2012 and December 2014. All procedures were performed at Department of Cardiac Surgery, University Hospital Ostrava, Czech Republic. Written informed consent was obtained from all patients prior surgery. Data were obtained from the National Registry of Cardiac Surgery, Czech Republic and were analysed retrospectively.

The patients suffered from structural heart disease, which was the basic indication for the heart surgery procedure, together with one type of atrial fibrillation (paroxysmal, persistent, or long-standing persistent), due to which left atrial cryoablation was performed as a part of the surgical procedure. Extent of left atrial ablation procedure is described below. Thirty-five patients (35%) from these population underwent concomitant mapping and radiofrequency ablation of ganglionated plexi (Group GP) whereas 65 patients (65%) had no ganglionated plexi intervention (Group LA). Both groups were comparable and no significant differences were observed in the monitored preoperative comorbidities, apart from the incidence of Stroke/Transitory Ischemic Attack (TIA) in the history of the patients. Preoperative data are presented in Table 1. The representation of individual types of atrial fibrillation in both groups was also comparable, and is presented in Table 2.

**Table 1:** Preoperative variables. Data are presented as mean ± standard deviation or as number of patients with percentages. TIA, transitory ischemic attack; NYHA, New York Heart Assotiation; CHADS-VASc, congestive heart failure, hypertenstion, age, diabetes, prior stroke, vascular disease history, age, sex; LV EF, left ventricular ejection fraction; LVED, left ventricular end-diastolic diameter

	Group GP *n* = 35	Group LA *n* = 65	*p* value
Age	69 ± 6.4	69 ± 7.4	0.617
Sex (M/F)	18/17 (51/49)	31/34 (48/52)	0.721
Body mass index	31 ± 4.9	30 ± 4.9	0.099
Hypertension	32 (91)	53 (81)	0.186
Diabetes mellitus	9 (26)	12 (18)	0.396
Creatinine	103 ± 45.4	102 ± 27.2	0.298
Stroke/TIA	2 (6)	14 (22)	0.040
Redo cardiac surgery	0	1 (2)	
NYHA	2	2	0.999
Euroscore II	4 ± 2.4	4 ± 4.7	0.301
CHA2DS2-VASc score	3.8 ± 1.4	3.6 ± 1.5	0.387
Structural cardiac disease			
Mitral valve disease	25 (71)	49 (75)	0.667
Tricuspidal valve disease	17 (49)	34 (52)	0.721
Aortic valvae disease	6 (17)	18 (28)	0.239
Coronary artery disease	21 (60)	32 (49)	0.303
Other	4 (11)	15 (23)	0.157
LV EF (%)	45 ± 10.2	50 ± 9.8	0.057
EF LK > 50%	15 (43)	42 (65)	
EF LK 30–50%	17 (49)	20 (31)	
EF LK < 30%	3 (8)	3 (4)	0.109
Left atrium diameter (mm)	45 ± 4.8	47 ± 5.5	0.544
LVED diameter (mm)	54 ± 6.7	51 ± 5.8	0.086

**Table 2:** Type of atrial fibrilation. Data are presented as number of patient with percentages

Type of atrial fibrilation	Group GP *n* = 35	Group LA *n* = 65	*p* value
Paroxysmal	14 (40)	28 (43)	
Persistent	15 (43)	24 (37)	
Longstanding persistent	6 (17)	13 (20)	0.838

### Surgical technique

**Fig. 1:**
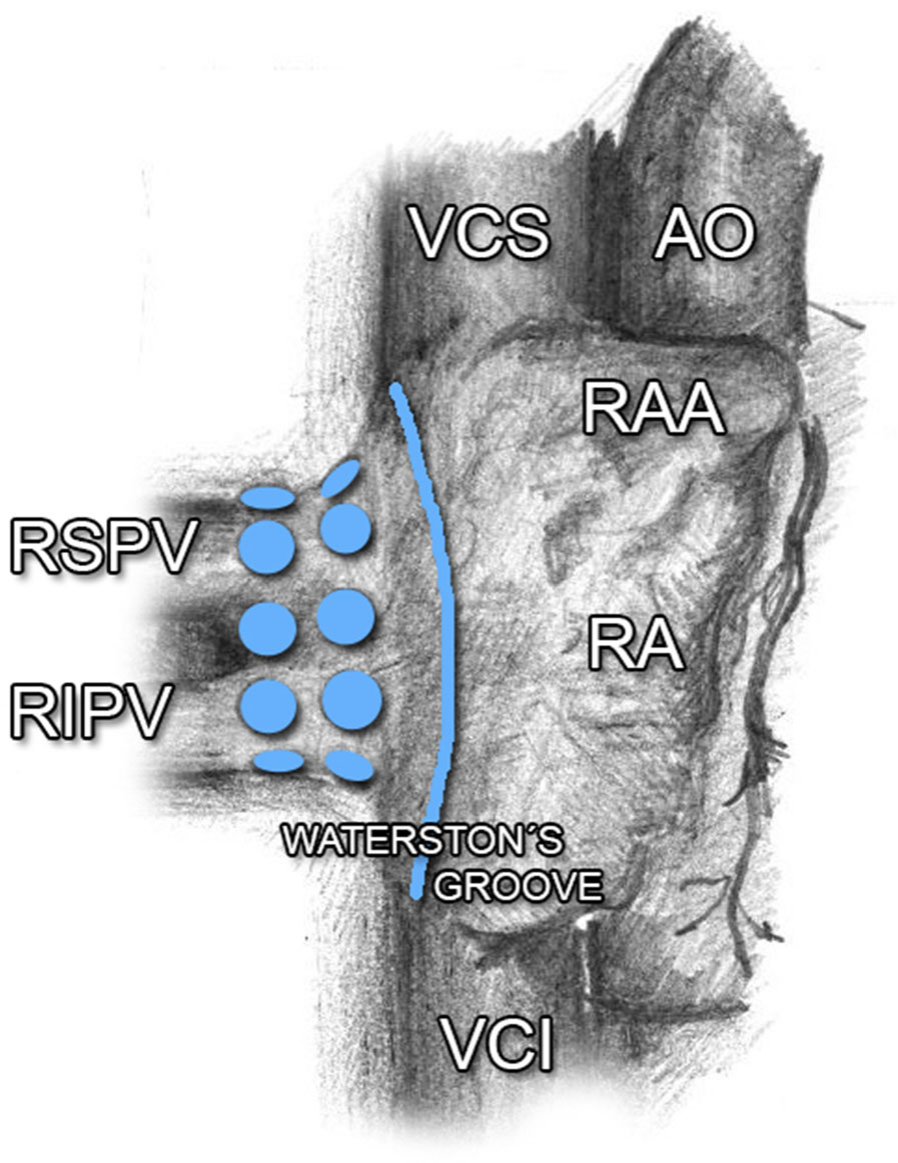
Ganglionated plexi around the orifice of right PVs. AO, aorta; PVs, pulmonary veins; RA, *right* atrium; RAA, *right* atrial appendage; RIPV, *right* inferior pulmonary vein; RSPV, *right* superior pulmonary vein; VCS, vena cava superior; VCI, vena cava inferior

**Fig. 2:**
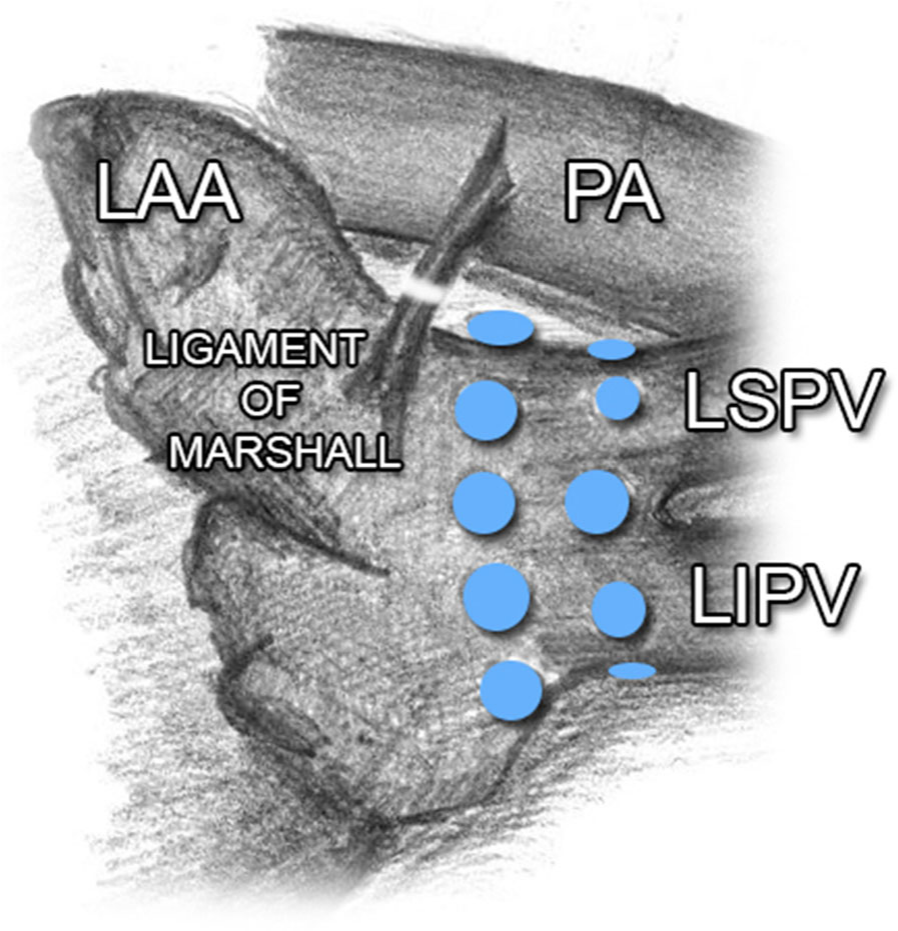
Ganglionated plexi around the orifice of left PVs. LAA, *left* atrial appendage; LIPV, *left* inferior pulmonary vein; *LSPV*, *left* superior pulmonary vein; PA, pulmonary artery; PVs, pulmonary veins

All patients underwent an open-heart surgery from median sternotomy, with the use of extracorporeal circulation, with bicaval cannulation and venting of arteria pulmonalis. In patients in the Group GP, mapping of GP around the orifice of pulmonary veins (PVs) (Fig. 1 and Fig. 2) was performed in the first stage, together with their radiofrequency (RF) ablation. In the area of right-side PVs, the procedure was performed prior to initiation of extracorporeal circulation (ECC). In case of the left-side PVs; the procedure was performed after initiation of ECC. Part of this procedure was also a discision and ablation of the ligament of Marshall.

**Fig. 3:**
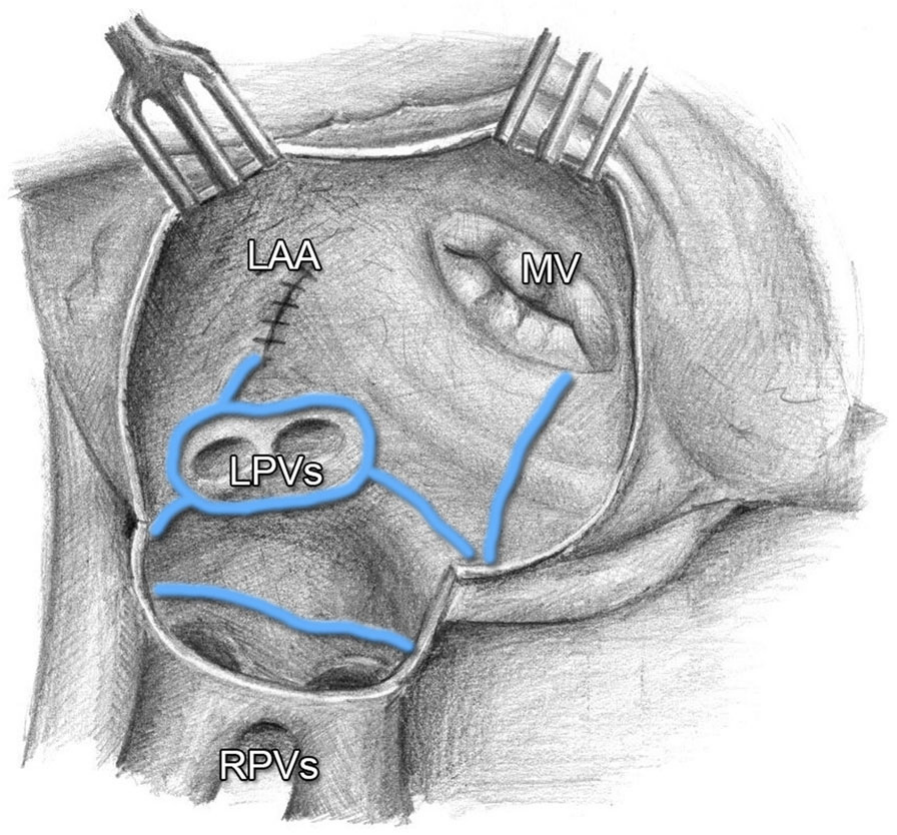
*Left* atrial cryoablation lesion set. LAA, *left* atrial appendage; MV, mitral valve; LPVs, *left* pulmonary veins; RPVs, *right* pulmonary veins

GP mapping was performed with “Isolator Multifunctional Pen” (AtriCure, West Chester, Ohio, USA), using high-frequency stimulation (1000 beats min¯^1^, potential 18 V, pulse width 1.5 ms), and PACE 203 H external stimulator (OSCOR Inc., Palm Harbor, USA). The indication for GP ablation was a doubling in the R-R interval in the sinus rhythm, or ventricular rate slowing of more that 50% [27] associated with a decrease of blood pressure > 20 mmHg in patients with AF [28]. In case of a positive response, radiofrequency ablation of the ganglia was performed following switching of the pen at the console. This procedure was repeated until the activity of the ganglia has disappeared. The following stage of the surgical procedure was identical for both groups of patients. After aortic cross-clamping and administration of cardioplegia, heart surgery procedure was performed, a part of which was the left atrial cryoablation in the extent of the right-side and left-side PVs isolation, box lesion (connecting right and left PVs lesions), lesion of the left atrium isthmus (connecting lesion with mitral annulus), resection of the left atrial appendage, and connecting lesion of the appendage base with left superior PV (Fig. 3). The procedure was performed with the Cardioblate Cryoflex probe (The Cardioblate CryoFlex Argon-powered Cryoablation System, Medtronic USA, Inc.). Lesions were created by temperature from −120 to −160 °C, administered for the period of 60 s [29, 30]. When the left atrium was opened, lesions were done endocavitally, and epicardially in other cases. The performed surgical procedures and duration of extracorporeal circulation are presented in Table 3.

**Table 3:** Main surgery procedures. Data are presented as mean ± standard deviation or as number of patients with percentages. MV, mitral valve; TV, tricuspidal valve; AV, atrial valve; CABG, coronary artery bypass graft; ECC, extracorporeal circulation

Group GP *n* = 35	Group LA *n* = 65		*p value*
MV repair	19 (54)	40 (62)	
MV replacement			
mechanical prosthesis	2 (6)	2 (3)	
biological prosthesis	4 (11)	7 (11)	0.863
TV repair	17 (49)	34 (52)	0.721
AV replacement			
mechanical prosthesis	1 (3)	2 (3)	
biological prosthesis	5 (14)	15 (23)	0.570
CABG	15 (43)	26 (40)	0.782
Other	4 (11)	15 (23)	0.157
Cross-clamp time	87 ± 26.4	97 ± 27.8	
ECC time	140 ± 41.4	135 ± 36.8	0.429

### Statistical analysis

In order to describe the patient file, basic descriptive statistics methods were used (median, arithmetical mean, standard deviation, frequency tables).

For quantitative variables, Shapiro-Wilk test of normality was used. Based upon data distribution, two-choice t-test, or non-parametric two-choice Wilcoxon test was used to compare the patient files. In order to test the difference of qualitative variables, chi-quadrate test was used, or Fisher’s exact test (in cases when it was not possible to use the chi-quadrate test).

In order to assess the parameters prior to the procedure and at 1 year after the procedure, paired t-test was used for quantitative variables, and symmetry test was used for qualitative variables. In order to obtain graphical representation, bar charts were used. The statistical tests were evaluated at the level of significance of 5%. The Stata programme, version 13 was used in order to process the statistical results.

The statistical analysis was performed at the Centre of Epidemiology Research, Faculty of Medicine, University of Ostrava.

### Postoperative management

The patients using anti-arrhythmic medication prior to the surgery procedure continued in taking the medication also in the post-operative period, unless the medication was contraindicated for any reason (e.g. serious heart rhythm disorders). In the therapy of postoperative AF, anti-arrhythmic medication Class Ic, II, III, IV and V was used (Vaughan Williams classification). The Class II (ß-blockers) was excluded from the assessment of frequency of the anti-arrhythmic medication in time of follow up. The anti-arrhythmic medication of the first choice was Amiodarone. Its saturation was initiated with intravenous infusion, with subsequent per oral medication use. Unless pharmacological version was performed, electrical cardioversion was performed in order to achieve the effective level of Amiodarone (> 6 g). In case of patients in whom administration of Amiodarone was contraindicated, other suitable anti-arrhythmic medication or combination of such was used. Anticoagulation medication (Warfarin) was administered in all patients after heart valve surgery. In patients using anticoagulation medication prior to the surgery for AF, administration of the medication continued also after the procedure. In patients with postoperative arrhythmia, Warfarin was administered in cases, when the arrhythmia lasted for the period exceeding 48 h. The dose of Warfarin was chosen according to the assessment of prothrombin time and the range of therapeutic level according to the International Normalized Ratio (INR); the length of duration was chosen according to the type of indication of the respective anticoagulation medication. The duration of therapy administered due to AF was 3 months. Following medication management was performed depending on the heart rhythm.

### Follow up

Assessment of the heart rhythm in patients was performed at 7 day, at 3 and 12 months after the surgical procedure. Heart rhythm was monitored continuously in all patients after the surgery. On the seventh postoperative day, evaluation of the heart rhythm was performed, using the record for the last 24 h. In the third and twelfth postoperative month, the patients were examined by a cardiologist in at our outpatient clinic. The assessment included collection of history, performance of physical examinations, 12-lead electrocardiography (ECG) and echocardiography (ECHO) examination. The assessment of heart rhythm was based on 24-h Holter monitoring, which was performed at our outpatient clinic, or by reffering cardiologists. Recurrent AF was defined as arrhythmia (AF, flutter, atrial tachycardia) lasting at least 30s. Patient medication was adjusted according to the findings of cardiology examination; also the possibility of performing electric cardioversion was considered, together with the need of implantation of permanent cardiostimulation.

## Results

### Postoperative results

No complications related to the ablation procedure were observed peroperatively or postoperatively. Overall 30-day mortality was 4%: one patient died in the Group GP (2.9%), three patients died in the Group LA (4.6%). The average EUROscore of the deceased patients was 4.01%.

The average length of hospitalization in our patient file was 15.7 days (Group GP 15 days, Group LA 16.3 days).

Due to serious postoperative complications, revisions were performed due to bleeding (*n* = 4), renal insufficiency requiring haemodialysis (*n* = 5), stroke/TIA (*n* = 1), sternal osteomyelitis (*n* = 1), Multiple Organ Dysfunction Syndrome (MODS) (*n* = 3), heart failure (*n* = 3), Acute Myocardial Infarction (AMI) (*n* = 1), respiratory insufficiency requiring re-intubation (*n* = 6), and bronchopneumonia (*n* = 8). These complications were observed in the total of 18 patients (Group GP 7 (20%), Group LA 11 (17%), *p* = 0.702). A higher accumulation (2 to 5) was noted in eight patients. In most cases, these patients died within 30 days.

### Follow up results

In the course of the 12-month follow up, one patient died in the Group GP, and one patient interrupted his contact with our outpatient clinic, two patients died in the Group LA. The final statistical analysis contains only the data from the patients, who remained in the study for whole follow-up period (Group GP *n* = 32, Group LA *n* = 60).

**Fig. 4:**
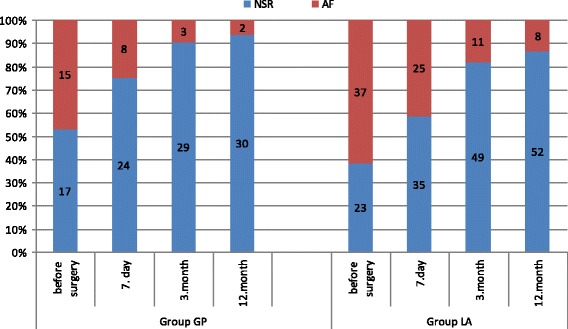
Incidence of normal sinus rhythm (NSR) and atrial fibrillation (AF)

**Fig. 5:**
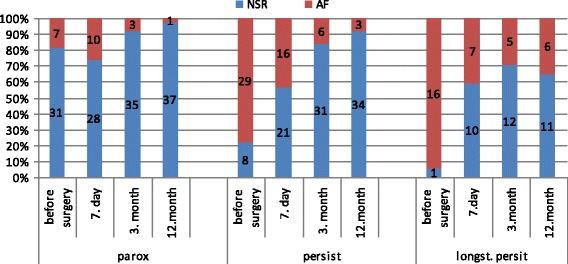
Incidence of normal sinus rhythm (NSR) and atrial fibrillation (AF) according to type of AF

The number of patients with NSR and AF in individual phases of follow-up, either in the subgroups – GP and LA, or in subgroups according to the type of fibrillation, is presented in Fig. 4. and Fig. 5.

The normal sinus rhythm (NSR) was achieved, regardless of the type of AF, in the course of the one-year follow-up, in 30 patients (94%) in the Group GP and in 82 patients (89%) in the Group LA (*p* = 0.485). Comparable results were also observed in both groups as regards the number of patients with NSR off anti-arrhythmic drugs (Group GP 16 (50%), Group LA 28 (47%), *p* = 0.184). Depending on the type of fibrillation at the end of follow-up, NSR was achieved in more than 90% of patients with paroxysmal and persistent fibrillation; the frequency of NSR was significantly lower in patients with long-standing persistent atrial fibrillation (97% paroxysmal, 91% persistent, 64% long-standing persistent, *p* = 0,003).

A total of 60 patients (63%) were on anti-arrhythmic medication at discharge (Group GP 18 (53%), Group LA 42 (68%)). In the sub-group of anti-arrhythmic medication, Amiodarone was the most frequently used drug (95%), the combination of Amiodarone and ß-blockers was also frequently observed (62%).

Stroke was observed in two patients in total (2.17%, *n* = 92). One patient suffered a stroke with a phatic disorder without lateralization in the perioperative period, with subsequent normalization of the neurological deficit. Another case of stroke was observed in one patient in the course of the follow-up period.

During follow-up, electrical cardioversion was performed in eight patients in the Group GP (23%), and 20 patients in the Group LA (31%) (*p* = 0.408). Eighty-five percent of cardioversions were performed during hospitalization after the surgery.

Permanent pacemaker was implanted in four patients from our file, already prior to the surgery. A total of eight patients (8%, *n* = 100) required an implantation of a permanent pacemaker in the postoperative period (3 patients in the Group GP (8.6%, *n* = 35), and 5 patients in the LA Group (7.7%, *n* = 65) (*p* = 1.000)). Serious heart rhythm disorders, resulting in implantation of a permanent pacemaker, included Sick Sinus Syndrome (SSS) in three cases, third-degree AV block (complete heart block) in two cases, junction rhythm, brady-form of AF, and asystole, always in one case each. In the sub-group of patients requiring pacemaker implantation in the postoperative period, a combined procedure at mitral and tricuspid valve was performed in six patients, revascularization of the myocardium (coronary bypass) in three patients, and aortal valve replacement in two patients. In one patient, implantation of epicardial electrode was performed in the course of the cardiac surgery procedure, due to findings of atrial fibrillation prior to the surgery. Planned implantation of Implantable Cardioverter Defibrillator (ICD) followed after the surgery. One patient required an implantation of permanent pacemaker due to brady-form of AF at 11 months following the surgical procedure. The resulting frequency of pacemaker implantation at the end of the follow-up period was 10%.

During further assessment, we investigated the relation between the recurrence of AF and certain pre-operative variables. The group with the parameter of left atrium size >50 mm included 22 patients with NSR (81%), and five patients with AF (19%) (*p* = 0.129); in the group of patients with mitral valvae disease, there were 60 patients with NSR (89%), and 7 patients with AF (11%) (*n* = 0.831); the group of patients with concomitant occurrence of mitral and tricuspid valvae defects contained 39 patients with NSR (87%), and 6 patients with AF (13%) (*p* = 0.458). No significant differences were found between the recurrence of AF and the above-listed variables.

In comparison of atrial fibrillation types was showed significantly higher AF recurrence in patients with long-standing persistent AF at the end 12-month follow up (paroxysmal 2.6%, persistent 8.1%, long-standing persistent 35.3%, *p* = 0.003).

### Limitations

In this report, we describe our early experiences with a mapping and ablation of GP in our low-volume center. The main limitations of our single-center study is retrospective analysis of data and relatively small number of patients, who underwent GP ablation. The left atrial cryoablation enhanced by mapping and ablation of GP (Group GP) was performed by only one surgeon, who was educated by proctor for Atricure. Although extreme caution was paid to proper mapping of GP, we cannot completely exclude the possibility of misdiagnosing the GP location. The posterior area of the left atrium isn’t accesible for this procedure through median sternotomy approach. The consideration for safety of the patient always prevails over procedural completeness. LA ablation in patients of control group (Group LA) was performed by other surgeons of our team with one type of cryoprobe.

The surgery procedures were performed in the course of 30 months, with subsequent 12-month follow-up.

The limiting factor for performance of ablation due to AF was the size of the left atrium ≤60 mm. Other factors, which also influenced the indication for ablation in some cases, included the duration of AF, extent of the surgical procedure, and haemodynamic condition of the patient prior to the surgery.

No statistically significant difference in pre-operative variables was observed between both compared groups, except for the incidence of Stroke/TIA in patient anamnesis. The assessment of heart rhythm was based on 24-h ECG monitoring.

## Discussion

In our study, we refer on our early experience with GP ablation enhancing the left atrium cryoablation in the course of surgical treatment of AF in patients undergoing cardiac surgery.

As we have already mentioned in the introduction, autonomic nervous system plays an important role in the initiation and maintenance of AF. Key role is played namely with ganglionated plexi, which mediate and modulate interactions between the extrinsic and intrinsic autonomic nervous system [10, 17], and namely plexi localized in the atrial walls. These plexi are concentrated in centres with maximum incidence around orifices of pulmonary veins [8, 17, 31–33].

Three key areas around the orifice of right-side PVs have been studied and presented in earlier publications: area between the right superior PV (RSPV) junction and LA, and superior vena cava (SVC) (dorsal right atrial subplexus) [9], medially from the right PV (RPVs) junction and LA, along interatrial groove (anterior right GP, ARGP) [8, 31] and between the inferior vena cava (IVC) orifice and the left atrium, in a close proximity of the coronary sinus orifice (the inferior area, inferior right GP, IRGP) [8, 31]. ARGP and IRGP play an important role in the regulation of SA and AV nodi [9, 12, 17, 27, 34]. Other two GP centres are located around the orifice of left-side PVs. These left-side centres manifest a significantly lower activity than the right-side GP [35, 36]. Ligament of Marshall has been reported as another independent centre, which may be the source of focal activity [37].

Although we did not keep a strict protocol regarding the frequency of activity and GP ablations in individual areas during our study, we observed, in concordance with previous studies, a significantly higher GP activity on the right side, namely in the area of RSPV junction – LA, behind SVC. Although the evidence regarding the influence of GP on the initiation and duration of AF is well known, the importance of GP ablation in the therapy of AF is not so clear, and studies, which have been performed so far, bring inconclusive evidence.

Experimental studies have proven influence of AF initiation in vagal denervation [38, 39], or chemical GP ablation [14]. In clinical practice, isolated GP ablation in the treatment of AF has been shown as insufficient [40, 41], and is not recommended [42]. Nevertheless, some authors support GP ablation as a part of a more complex catheterization [21, 22] or surgical ablation [24, 43–45]. In this relation, work of Pappone et al. [21] is frequently cited; the authors achieved 99% success rate (freedom from AF) in the course of 12-month follow-up in patients with paroxysmal AF, in whom catheterization RF ablation was performed – circulation of PVs with a complete vagal denervation (= interruption of all vagal reflexes around PVs orifice).

In surgery, GP ablation is most frequently used as an enhancement of ablation therapy of AF alone performed from mini-invasive aproache, with or without the use of extracorporeal circulation, from bilateral thoracotomy [45, 46], or via fully thoracoscopic approach [24, 43, 44]. These studies present very good results achieved in the treatment of paroxysmal, persistent and long-standing persistent AF. However, most of these studies are retrospective, non-randomized clinical trials. There also exist works speaking against the use of GP ablation, presenting good results of AF treatment with ablation therapy only, without the use of GP ablation [47, 48]. In these cases, certain role may have been played by the already mentioned GP topography. Most of the plexi are located in areas of ablation lines; that is why it is possible to presume that they will be destroyed (eliminated) already in the course of a standard ablation, without a targeted GP mapping. This theory is also supported by the findings of McClelland et al. [46]. The authors claim that following circumferential isolation of pulmonary veins with bipolar RF, the GP activity was limited by 79%. Gelsomino et al. [49] use this theory to explain the failure to demonstrate the benefit of GP ablation in their study. This study was published last year, and similarly to our study, deals with the importance of GP ablation in patients with concomitant atrial fibrillation in the course of open-heart surgery. The authors compared groups of patients with persistent and long-standing persistent AF, in whom radiofrequency ablation was performed, including (Group 1), or not including (Group 2) GP ablation. The average follow-up period was 36.7 months. When comparing both groups, no difference was observed in the percentage of patients with NSR on-AAD (anti-arrhythmic drugs) (86.4% vs. 79.7%, *p* = 0.07), or in the percentage of patients with NSR off-AAD at the end of the follow-up period (75.5% vs. 67.8%, *p* = 0.08).The performed risk analysis showed identical incidence of AF recurrence in both groups. Also the group of Alex et al. [50] was unsuccessful in their attempt to influence the incidence of AF in patients after CABG (coronary revascularization) procedure with heart denervation.

Other reasons standing in opposition of the performance of targeted GP ablation are the findings in the area of restoration of the heart autonomic system. The sympathetic re-innervation was described by Kaye et al. [51] already in 1993, in patients following heart transplantation. It is possible to object that in cases of heart transplantation, neuronal fibres are only interrupted, and direct destruction of neurons of the heart neural system is not performed. However, newer experimental studies bring clear evidence regarding re-innervation also following radiofrequency parasympathetic denervation (vagal denervation). Tests were also performed on canine hearts, the samples were monitored for the period of four weeks following ablation on average [52, 53]. Similar results of neural function restitution have also been presented regarding experimental demonstration of administration of cryoenergy on the neural tissue. After administration of cryoenergy, the function of sensory and motor nerves is restored within several months [54–56]. This work cites only selected publications regarding GP ablation. Nevertheless, inconclusiveness of results and conclusions presented in these studies points towards the need of further research in the form of randomized, prospective clinical trials.

The issue of long-term efficacy of GP ablation has been discussed on numerous occasions and conclusion is not clear. On the basis of our early experiences and of recent experiences of other colleagues from cardiac centres we decided disrupt GP ablation performance and we are waiting for results of following studies. Although our team was educated by Atricure promotor, we performed GP ablation in only 35 patients and the learning curve may play role determinig our negative result.

A frequent topic of discussions regarding the surgical treatment of AF is the extent of the lesion set. This topic was analysed in the meta-analysis performed by Barnett et al. [57]; the results of this analysis show that surgical biatrial ablation (range, 92.0%-87.1%) in the analysed studies was more effective in the treatment of AF than the procedure limited to the left atrium (range, 86.1-73.4%). Also the results of comparing various extent of ablation in the above cited work of the Italian authors [49] are in accordance with this finding; the authors concluded that biatrial ablation, as well as compete left atrial lesion set are suitable for improving rhythm outcomes, however, on more detailed analysis, a significantly lower incidence of AF recurrence was observed in patients with biatrial ablation when compared to the patients with left atrium ablation (18.5 vs. 36.5%, *p* = 0.001), and absence of RA lesions (right atrium ablation) was determined as one of the predictors of AF recurrence.

In our present, as well as previous study [58], we managed to achieve similar good outcomes with performance of left atrium ablation in achievement and duration of NSR in the group of patients with paroxysmal and persistent AF. However, in the group of patients with long-standing persistent, or permanent AF, we observed a significantly higher AF recurrence, which increases with the period of follow-up.

These results and conclusions made us re-evaluate the extent of the ablation procedure performed at our centre, namely in patients with persistent and long-standing persistent AF, and enhancing the procedure with ablation of RA lesions (Cox-MAZE IV procedure), the performance of which is aimed at the period of reperfusion, and thus should not have any influence on the cross-clamp time, the pump time, or the overall time of surgery [59]. Currently the Cox MAZE IV without GP ablation is preferred approach to treatment of concomitant AF in patients with persistent AF and mitral valve diseases at our centre.

Permanent pacemaker implantation is a known complication following heart surgery procedures. Its incidence reported in the literature reaches 1–11% [60–65], and is comparable for heart surgeries with and without ablation. Elahi et al. [62] reported in their study of patients undergoing heart valve surgery the frequency of permanent cardiac stimulation of 6%, and the identified predictors for implantation include reoperation, cross-clamp time, combined procedure on heart valves and absence of NSR prior to the surgical procedure.

Low values were also reported by Merin et al. [63] (1.4%), with a higher incidence in patients following aortic valve replacement (5.7%), and the team of Gelsomino et al. [49] (0.6% in the control group of patients undergoing cardiac surgery, with a various extent of ablation lesions set due to AF). On the other hand, there exist publications reporting the frequency of permanent cardiac stimulation around 20% [66, 67]. In case of the study presented by Prasad et al. [66] the study subjects were patients after cardiac surgery with associated ablation due to AF, in most of whom sick sinus syndrome was diagnosed already prior to the surgery. In the group of patients with a normal function of the sinus node, the reported rate of implantation reached 8%. In our patient group, a total of eight patients required permanent implantation (8%) in the postoperative period (until discharge). At the end of the 12-month follow-up, the overall rate of implantation reached 10%.

Left atrium appendage (LAA) elimination in patients with AF is performed due to the risk of thromboembolic complications. Dawson et al. [68] presented the results of studies dealing with this topic in his summary work. Although the benefit of LAA elimination was not confirmed, we perform this procedure in all patients with AF undergoing cardiac surgery. The procedure may be performed safely, without the need of prolongation of the overall time of surgery and increasing of the risk of perioperative bleeding [69]. As far as the surgical technique is concerned, use of instruments designed specifically for appendage occlusion, rather than use of staplers or the cut-and-sew technique is recommended [70]. Ligation or suture of the appendage is not recommended due to a high incidence of recanalization [71, 72]. Very good results have been published regarding the use of AtriClip device (Atricure, Inc., Westchester, OH, USA); use of this device leads not only to an ideal LAA occlusion and prevention of stroke but also to achieving electrical isolation of LAA and reduced AF recurrence [73].

At present, we use at our centre the technique cut-and-sew for removal of LAA. The incidence of thromboembolic complications (stroke) among the patient population in our present study reaches approx. 2.2% at the end of one-year follow-up. In the previous study [58] performed at our centre, the incidence of thromboembolic events was 4.6%, or 5.9%, at the end of one-year and two-year follow-up, respectively. This corresponds with the frequency of thromboembolic events in patients after surgical ablation presented in published studies and results summarized in the meta-analysis by Gray et al. from 2011 [74].

The size of the left atrium is one of the factors influencing the AF recurrence. Gillinov et al. [61]*,* in their study present a significantly higher AF recurrence in patients with the LA diameter of 60 mm (15%), in comparison with the LA size >40 mm. Damiano et al. [75] present up to 50% AF recurrence in LA diameter > 80 mm et LA reduction procedures presented in this study were not successful in the prevention of recurrence.

Upon analysis of our patient group, we did not observe any significant dependency of AF recurrence on the LA diameter in patients with LA > 50 mm. This result may have been influenced by the relatively small number of study subjects and the fact that our patient file did not include any patients with LA > 60 mm undergoing surgery; however, one patient in the GP Group had LA diameter of 63 mm.

## Conclusion

We have not proven an improvement of results of left atrial cryoablation enhanced with a targeted RF ablation of GP in the AF treatment in patients undergoing cardiac surgery. Mapping and targeted GP ablation is not performed at our centre in other patients at the moment, as we are waiting for results of randomized controlled trials.

Cryoablation of the left atrium is a reasonable treatment of choice in patients with paroxysmal and persistent AF. In case of patients with long-standing persistent AF, higher recurrence of AF has been observed.

## Abbreviations

AAD: Anti-arrhythmic drugs
AF: Atrial fibrillation
AIM: Acute myocardial infarction
ANS: Autonomic nervous system
ARGP: Anterior right ganglionated plexi
AV: Atrioventricular
CABG: Coronary artery bypass graft
ECC: Extracorporeal circulation
ECG: Electrocardiography
ECHO: Echocardiography
GP: Gangionated plexi
ICD: Impantable cardioverter defibrillator
INR: Internal normalized ratio
IRGP: Inferior right ganglionated plexi
IVC: Inferior vena cava
LA: Left atrium
LAA: Left atrial appendage
MODS: Multiple organ dysfunction syndrome
NSR: Normal sinus rhythm
PVs: Pulmonary veins
RA: Right atrium
RF: Radiofrequency
RPVs: Right pulmonary veins
RSPV: Right superior pulmonary vein
SA: Sinoatrial
SSS: Sick sinus syndrome
SVC: Superior vena cava
TIA: Transitory ischemic attack
